# 424. The Impact of COVID-19 Response on Infection Prevention Programs and Practices in Southeastern United States

**DOI:** 10.1093/ofid/ofab466.624

**Published:** 2021-12-04

**Authors:** Sonali D Advani, Sonali D Advani, Andrea Cromer, Brittain A Wood, Esther Baker, Kathryn L Crawford, Linda Crane, Linda Roach, Polly W Padgette, Elizabeth Dodds Ashley, Ibukunoluwa Akinboyo, David J Weber, David J Weber, Emily Sickbert-Bennett, Deverick J Anderson

**Affiliations:** 1 Duke University School of Medicine, Duke Infection Control Outreach Network, Durham, NC; 2 Duke Infection Control Outreach Network (DICON), Inman, SC; 3 Duke Infection Control Outreach Network, Durham, NC; 4 Duke Center for Antimicrobial Stewardship and Infection Prevention, Durham, NC; 5 Duke University, Durham, NC; 6 University of North Carolina, Chapel Hill, NC; 7 UNC Health Care, Chapel Hill, NC

## Abstract

**Background:**

Early assessments of COVID19 preparedness reported resource shortages, use of crisis capacity strategies, variations in testing, personal protective equipment (PPE), and policies in US hospitals. One year later, we performed a follow-up survey to assess changes in infection prevention practice and policies in our diverse network of community and academic hospitals.

**Methods:**

This was a cross-sectional electronic survey of infection preventionists in 58 hospitals within the Duke Infection Control Outreach Network (community) and Duke/UNC Health systems (academic) in April-May 2021 to follow-up our initial survey from April 2020. The follow-up survey included 26 questions related to resource availability, crisis capacity strategies, procedures, changes to PPE and testing, and staffing challenges.

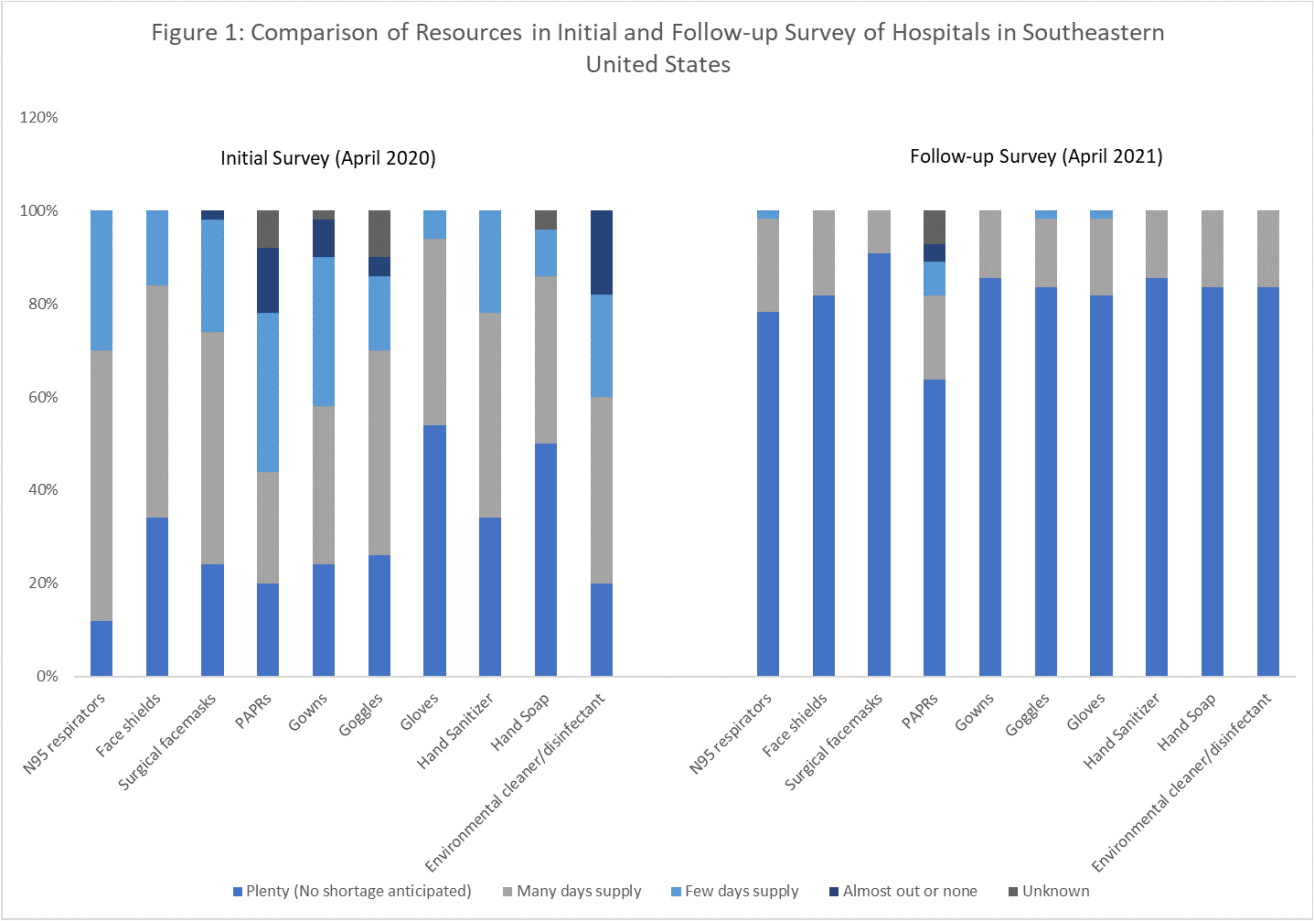

**Results:**

We received 54 responses (response rate, 93%). Facilities reported significantly fewer PPE and resource shortages in the follow-up survey compared to our initial survey (Figure 1, P< 0.05). Only 32% of respondents were still reprocessing N95 respirators (compared to 73% in initial survey, P< 0.05). All hospitals performed universal masking, universal symptom screening on entry, and 30% required eye protection. In 2020, most hospitals suspended elective surgical procedures in March-April, and restarted in May-June. Approximately 92% reported in-house testing for SARS-COV-2 by April 2020, at least a third of which had a weekly capacity of >100 tests. Almost 80% performed universal pre-operative testing, while 61% performed universal preadmission testing for SARS-COV-2. Almost all hospitals switched from test-based to time-based strategy for discontinuing isolation precautions, majority in August-September 2020. Twenty-five percent hospitals reported infection prevention furloughs, staffing cuts, and or reassignments, while 81% reported increased use of agency nursing during the pandemic.

**Conclusion:**

Our follow-up survey reveals improvement in resource availability, evolution of PPE guidance, increase in testing capacity, and burdensome staffing changes. Our serial surveys suggest increasing uniformity in infection prevention policies, but also highlight the increase in staff turnover and infection prevention staffing shortages.

**Disclosures:**

**Sonali D. Advani, MBBS, MPH**, Nothing to disclose **David J. Weber, MD, MPH**, **PDI** (Consultant)

